# GASTRIC PLICATION ASSOCIATED WITH FUNDOPLICATION IN INDIVIDUALS WITH CLASS I OBESITY AND GASTROESOPHAGEAL REFLUX DISEASE: WEIGHT LOSS OUTCOMES, REFLUX-RELATED SYMPTOMS, ENDOSCOPIC AND pH MONITORING FINDINGS

**DOI:** 10.1590/0102-672020230033e1751

**Published:** 2023-07-17

**Authors:** Victor Kenzo Ivano, Marcio Apodaca-Rueda, Victor Kenichi Morisawa, Vinícius Basso Preti, Letícia Miyashiro, Everton Cazzo, Flavio Heuta Ivano

**Affiliations:** 1 Universidade Estadual de Campinas, Department of Surgery – Campinas (SP), Brazil; 2 Pontifícia Universidade Católica do Paraná, Department of Surgery – Curitiba (PR), Brazil; 3 Universidade Positivo, Faculty of Medicine – Curitiba (PR), Brazil.

**Keywords:** Bariatric surgery, Obesity, Gastroesophageal reflux, Esophagitis, Weight loss, Cirurgia bariátrica, Obesidade, Refluxo gastroesofágico, Esofagite, Redução de peso

## Abstract

**BACKGROUND:**

The association of gastric plication with fundoplication is a reliable option for the treatment of individuals with obesity associated with gastroesophageal reflux disease.

**AIMS:**

To describe weight loss, endoscopic, and gastroesophageal reflux disease-related outcomes of gastric plication with fundoplication in individuals with mild obesity.

**METHODS:**

A retrospective cohort study was carried out, enrolling individuals who underwent gastric plication with fundoplication at a tertiary private hospital from 2015–2019. Data regarding perioperative and weight loss outcomes, endoscopic and 24-hour pH monitoring findings, and gastroesophageal reflux disease-related symptoms were analyzed.

**RESULTS:**

Of 98 individuals, 90.2% were female. The median age was 40.4 years (IQR 32.1–47.8). The median body mass index decreased from 32 kg/m^2^ (IQR 30,5–34) to 29.5 kg/m^2^ (IQR 26.7–33.9) at 1–2 years (p<0.05); and to 27.4 kg/m^2^ (IQR 24.1–30.6) at 2–4 years (p=0.059). The median percentage of total weight loss at 1–2 years was 7.8% (IQR −4.1–14.7) and at 2–4 years, it was 16.4% (IQR 4.3–24.1). Both esophageal and extra-esophageal symptoms showed a significant reduction (p<0.05). A significant decrease in the occurrence of esophagitis was observed (p<0.01). The median DeMeester score decreased from 30 (IQR 15.1–48.4) to 1.9 (IQR 0.93–5.4) (p<0.0001).

**CONCLUSIONS:**

The gastric plication with fundoplication proved to be an effective and safe technique, leading to a significant and sustained weight loss in addition to endoscopic and clinical improvement of gastroesophageal reflux disease.

## RESUMO

**RACIONAL:**

A associação de plicatura gástrica com fundoplicatura é uma opção atrativa para o tratamento de indivíduos com obesidade associada à doença do refluxo gastroesofágico.

**OBJETIVOS:**

Descrever a evolução ponderal, clínica, endoscópica e de pHmetria após a gástrica com fundoplicatura em indivíduos com obesidade grau I e doença do refluxo gastroesofágico.

**MÉTODOS:**

Um estudo de coorte histórica foi realizado, incluindo indivíduos que realizaram gástrica com fundoplicatura em um hospital privado terciário de 2015 a 2019. Os dados sobre os resultados perioperatórios e de perda de peso, achados endoscópicos e de pHmetria de 24 horas e sintomas relacionados à doença do refluxo gastroesofágico foram analisados.

**RESULTADOS:**

Dos 98 indivíduos, 90,2% eram do sexo feminino. A idade mediana foi de 40,4 (IQR 32,1–47,8) anos. A mediana do índice de massa corporal diminuiu de 32 (IQR 30,5–34) kg/m^2^ para 29,5 (IQR 26,7–33,9) kg/m^2^ em 1–2 anos (p<0,05); de 2 a 4 anos, foi de 27,4 (IQR 24,1–30,6) kg/m^2^ (p=0,059). A mediana da porcentagem da perda total de peso em 1-2 anos foi de 7,8% (IQR −4,1–14,7) e em 2-4 anos, foi de 16,4% (IQR 4,3–24,1). Tanto os sintomas esofágicos quanto os extras esofágicos apresentaram redução significativa (p<0,05). Observou-se diminuição significativa na ocorrência de esofagite (p<0,01). A pontuação mediana do escore de DeMeester diminuiu de 30 (IQR 15,1–48,4) para 1,9 (IQR 0,93–5,4) (p<0,0001).

**CONCLUSÕES:**

A associação entre plicatura gástrica e fundoplicatura laparoscópica mostrou-se uma técnica eficaz e segura, levando a perda de peso significativa, bem como melhora endoscópica e clínica da doença do refluxo gastroesofágico.

## INTRODUCTION

Gastroesophageal reflux disease (GERD) and obesity are frequently associated, and GERD symptoms are usually worsened in this situation, which may increase the risk of developing severe esophagitis and pre-cancerous abnormalities^1^. Surgical treatment is a viable option for individuals refractory to continuous or intermittent drug treatment. It classically consists of total fundoplication associated with hiatal hernia repair, leading to more than an estimated 90% improvement in symptoms^4,7^. Nevertheless, the effectiveness of the treatment is compromised in individuals with overweight or obesity^14^.

Laparoscopic gastric plication (GP) is an evolving technique that has the potential to become an alternative to obesity treatment. As main advantages, in contrast to established techniques, it does not require foreign bodies (e.g., bands), or gastric resection (as in sleeve gastrectomy and anastomoses), and does not cause malabsorption of nutrients (as in gastric bypass and biliopancreatic diversion)^16^. In addition, its cost is lower because it does not require the use of surgical staplers or bands, or a long hospital stay. Few complications have been reported since this technique is a reversible and more conservative strategy than mainstream bariatric operations, especially if aiming at more modest weight loss^17^. The association of GP with fundoplication (GP-FP) is an attractive option for the treatment of obesity and GERD. It is reportedly a low-cost and highly reproducible surgical technique that does not rely on technological resources and/or sophisticated devices, and that aims at maximizing the co-joint benefits of fundoplication and gastric restriction in individuals with obesity and GERD^10,11,13,18^.

Individuals with mild obesity and refractory GERD are considered the ideal audience for such a procedure, since they do not present with the usual indications for conventional bariatric surgery currently established by the National Institutes of Health (NIH) criteria, and may also require surgical treatment for GERD^18^. Thus, considering the promising advantages of laparoscopic GP-FP, this study aims at describing the weight loss, endoscopic, and GERD-related outcomes of this surgical technique in this population.

## METHODS

### Study design

A retrospective cohort study was conducted by enrolling individuals who underwent GP-FP at the Hospital Sugisawa in Curitiba (PR), a tertiary private hospital, from April 2015 to April 2019. Data regarding the perioperative, weight loss, endoscopic findings, and GERD-related outcomes were analyzed. Individuals who underwent the procedure were called up by phone by members of the research team. Data were accessed from medical records, hospital spreadsheets and institutional databases and were analyzed. All individuals had undergone regular postoperative clinical evaluations other than those in this study, but they were not systematically performed for study purposes.

The individuals were twice evaluated (before and after surgery). Postoperatively, they were divided in two groups according to the time elapsed from surgery: 1–2 years and 2–4 years.

The study protocol was evaluated and approved by the Ethics Committee of Pontifícia Universidade Católica do Paraná, Curitiba (PR) (under 43520921.0.0000.0020/PUC-PR).

### Study population

Surgery was indicated for individuals of any gender, aged 18 to 70 years, who presented with a pre-operative body mass index (BMI) between 30 and 34.9 kg/m^2^ (class I obesity) and who had refractory symptoms and/or endoscopic findings consistent with GERD. Refractory GERD was defined as the presence of persistent troublesome GERD symptoms and objective evidence despite optimized proton-pump inhibitor (PPI) therapy, which consists of double-dose PPI therapy over at least eight weeks^19^. Individuals who did not comply with postoperative follow-up for at least one year, those who had already undergone other bariatric or anti-reflux operations, and those who did not opt to take part in the study were excluded.

Of 153 medical records analyzed in the period, 98 individuals met the inclusion criteria. The main reasons for the exclusion were follow-up of less than one year (n=35) and unacceptance to take part in the study (n=20).

Postoperatively, there were 35 individuals (35.7%) evaluated between 1–2 years, and 63 (64.3%) evaluated from 2–4 years.

### Assessment of gastrointestinal symptoms

Participants were assessed according to symptoms using the validated Gastrointestinal Symptom Rating Scale (GSRS) questionnaire^15^. The frequencies of esophageal and extraesophageal symptoms were evaluated. Esophageal symptoms included acid reflux and heartburn, and extra-esophageal symptoms were cough, hoarseness, abdominal distension, diarrhea, and flatulence.

### Anthropometric, endoscopic, and 24-hour pH monitoring data

Data regarding pre- and postoperative BMI, esophagogastroscopies, and 24-hour pH monitoring were acquired through electronic medical records, considering the baseline weight observed in the last pre-operative consultation.

All esophagogastroscopies were performed by the same team at the same facility. Erosive esophagitis was classified according to the Los Angeles criteria in grade A (mucosal breaks <5 mm), grade B (mucosal breaks =5 mm), grade C (coalescing mucosal breaks that affect <75% of the esophageal circumference), and grade D (coalescing mucosal breaks affecting ≥75% of the esophageal circumference)^5^.

Data regarding DeMeester scores were obtained from 24-hour pH monitoring performed by the same team. These scores were calculated by the sum of points according to the following characteristics, considering reflux as pH<4: number of 24-hour reflux episodes; number of reflux episodes that lasted more than 5 minutes; duration of the most prolonged reflux episode; percentage of total time with reflux; percentage of time with orthostatic reflux; percentage of time with supine reflux. The threshold value considered for the pathologic gastroesophageal reflux was 14.7^9^.

### Operative technique

**Figure 1:**
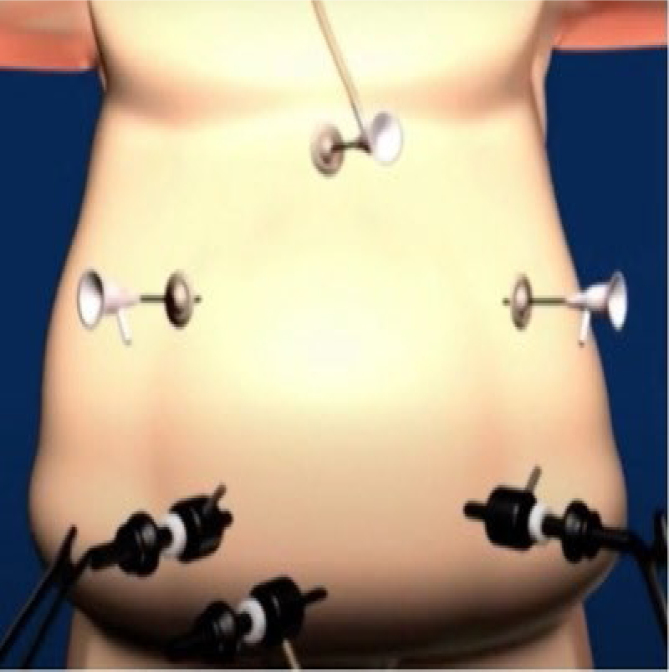
Trocars placement.

**Figure 2:**
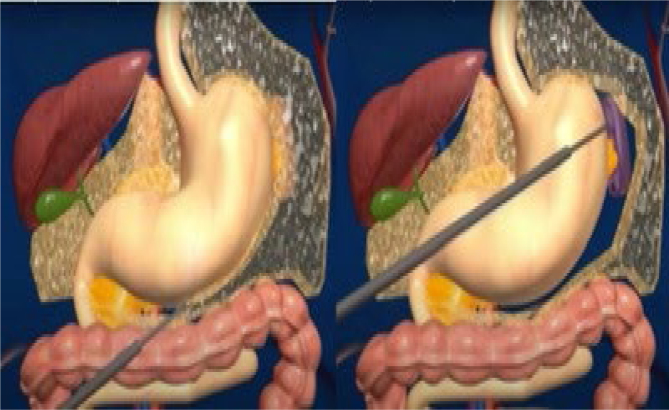
Ligation of the vessels of the greater curvature with ultrasound scalpel.

**Figure 3:**
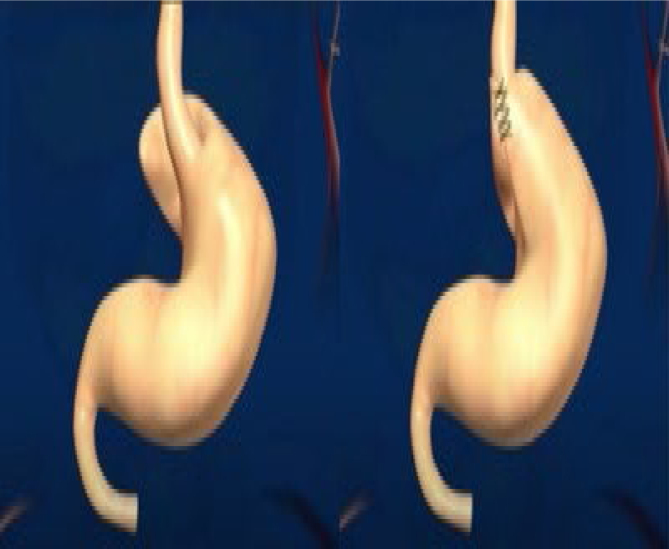
Nissen-Rossetti fundoplication.

**Figure 4:**
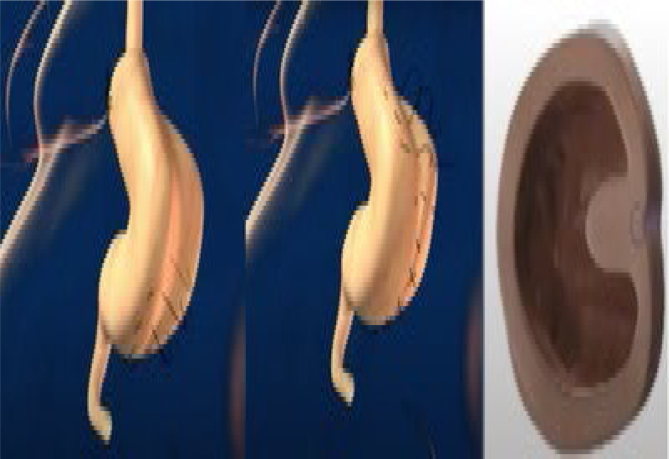
First suture with separate stitches and folding of the stomach. The third image shows a cross-section of the final aspect of this suture.

**Figure 5:**
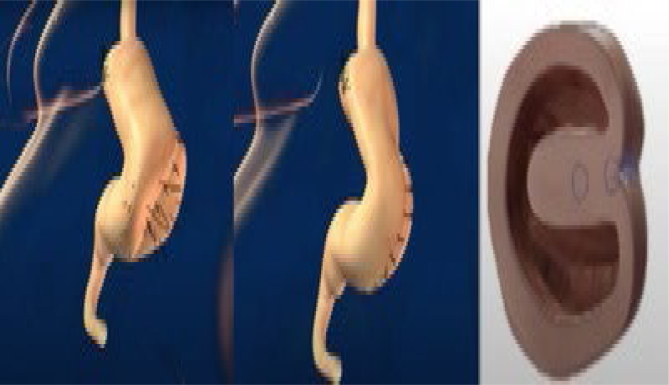
Second suture (continuous stitches) and imbrication of the first folding. The third image shows a cross-section of the final aspect of the suture.

The operations were performed laparoscopically. The trocars were placed as shown in Figure 1. There were two 12-mm trocars placed below both rib cages; the remaining were 5-mm trocars. Vessels in the greater curvature of the stomach and short vessels were divided with an ultrasound scalpel up to the angle of His (Figure 2). The lesser gastric curvature was then opened, and the abdominal esophagus was isolated for 3–5 cm. Subsequently, the right and left branches of the diaphragmatic crus were dissected and, when necessary, a hiatal hernia was pulled down. Hiatal hernia repair was conducted through a primary posterior suture of the crura using interrupted stitches with non-absorbable threads. The fundus was wrapped around the esophagus to complete the standard 360-degree Nissen-Rossetti fundoplication, performed through four interrupted stitches with non-absorbable threads. (Figure 3). Finally, the imbrication of the greater gastric curvature was performed through a longitudinal GP using four to five interrupted seromuscular sutures with non-absorbable threads starting from below the Nissen-Rossetti's wrap to the distal antrum (about 3–4 cm from the pylorus) to create a double-layer plication (inner interrupted and outer continuous) (Figures 4 and 5). The operation was entirely performed with a 32-Fr Faucher bougie inside the stomach.

### Statistical analysis

Normality was assessed through the Kolmogorov-Smirnov test. Continuous data were expressed through medians and interquartile ranges (IQR). To compare pre- and postoperative results, the *t*-test was used for paired data, and the Wilcoxon test was used for non-parametric data. A sub-analysis comparing BMI and the incidence of symptoms in earlier (1–2 years), and later (2–4 years) postoperative periods was also performed. The level of significance adopted was 5% (p<0.05). The analyses were performed using the software IBM SPSS Statistics for Windows 20.0 (IBM Corp., Armonk, NY).

## RESULTS

Of the 98 individuals included in the study, 90.2% were female. The median age was 40.4 years (IQR 32.1–47.8). The median pre-operative BMI was 32.0 kg/m^2^ (IQR 30,5–34); at 1–2 years, it was 29.5 kg/m^2^ (IQR 26.7–33.9); and at 2–4 years, it was 27.4 kg/m^2^ (IQR 24.1–30.6). The median BMI was significantly lower at 1–2 years than at baseline (p<0.05); and there were no significant changes between then and from 2–4 years on (p=0.059) (Table 1). Median percentage of total weight loss (TWL) at 1–2 years was 7.8% (IQR −4.1–14.7), and at 2–4 years, it was 16.4% (IQR 4.3–24.1).

**Table 1:** Anthropometric, endoscopic, pH monitoring, and clinical evolution of the study population.

		Baseline	1–2 years	2–4 years	p-value
n		98	35	63	NA
BMI (kg/m^2^)		32.0 (IQR 30.5–34)	29.5 (IQR 26.7–33.9)	27.4 (IQR 24.1–30.6)	<0.0001* BL vs. 1–2 years: p<0.01 BL vs. 2–4 years: p<0.01
Symptoms					
	Asymptomatic (%)	7.1	91.4	66.7	<0.0001
	Esophageal (%)	44.9	0	0	<0.0001
	Extra-esophageal (%)	6.1	8.6	30.2	0.001
	Esophageal and extra-esophageal (%)	41.8	0	1.6	<0.0001
Endoscopic findings					
	n	98	20	NA	NA
	No abnormalities (%)	0	55	NA	<0.0001
	Grade A esophagitis (%)	58.2	45	NA	1.00
	Grade B esophagitis (%)	41.8	0	NA	0.01
24-hour pH monitoring					
	n	48	14	NA	NA
	DeMeester score	30 (IQR 15.1–48.4)	1.9 (IQR 0.93–5.4)	NA	<0.0001

n: number of individuals; BMI: body mass index; IQR: interquartile range; BL: baseline; NA: not applicable

Regarding the symptoms, 92.9% of the patients presented with gastrointestinal symptoms before surgery, of which 44.9% were esophageal and 41.8% were esophageal associated with extraesophageal. At 1–2 years after surgery, 91.4% were asymptomatic and 8.6% had only extra-esophageal symptoms, such as flatulence or abdominal distention. In the period between 2–4 years after surgery, 66.7% remained asymptomatic, with a predominance of extra-esophageal symptoms (30.2%). All categories showed a significant reduction when compared to pre-operative symptoms (p<0.05) (Table 1).

As for the preoperative endoscopic findings, 58.2% presented with grade A esophagitis and 41.8% with grade B; no patient presented with grades C or D (Table 1). After surgery, 20 individuals underwent an esophagogastroscopy between 1–2 years; of these, 55% of the patients had no endoscopic abnormalities, and 45% had grade A esophagitis. Despite the low number of pos-operative examinations, a decrease in the occurrence and severity of esophagitis was observed after surgery (p<0.01). Regarding specific GP-FP-related findings in postoperative endoscopic evaluation, there was one case of cranial fundoplication migration (5%) and one case of undone gastric plication (5%).

In 48 individuals who underwent pre-operative pH monitoring, the median DeMeester score was 30 (IQR 15.1–48.4); after surgery, 14 individuals were evaluated, and the median score was 1.9 (IQR 0.93–5.4). The difference was statistically significant (p<0.0001) (Table 1).

There was no perioperative morbidity or mortality. No individual had dysphagic symptoms or difficulty in dieting. Two participants were reoperated during the later follow-up. One of them because of an unrelated cause (intestinal ischemia) four years after the procedure.

The other was reoperated after four years because of insufficient weight loss, undergoing a combined Nissen-sleeve gastrectomy with preservation of the previous Nissen's wrap. This technique was chosen because of the relatively low BMI of the patient to propose a gastric bypass (<35 kg/m^2^) without comorbidities alongside the absence of endoscopic and clinical esophagitis after the first operation.

## DISCUSSION

Surgical treatment plays an important role in the management of GERD as an alternative for individuals who do not wish to maintain continuous use of medications or in situations of refractoriness and high risk of complications, such as strictures and evolution to cancer. Nissen-Rossetti fundoplication associated with hiatal hernia repair is the standard technique, with a 90% improvement in symptoms^1,3^. However, in situations of overweight, obesity, or increased abdominal volume and pressure, the benefits of this procedure are limited, and even the risk of fundoplication migration is significant. Routinely performed bariatric surgical techniques, especially Roux-en-Y gastric bypass, in individuals with at least moderate obesity, are well-established treatment modalities for obesity-associated GERD, with strong evidence of benefits in the literature. However, individuals with overweight and mild obesity are not suitable for bariatric surgery according to current National Institute of Health (NIH) criteria and comprise a large population in which the Nissen operation has relevant limitations and leads to modest results. Thus, alternative proposals are needed for this group of individuals^3^.

GP is a restrictive surgical method, which consists of reducing the intragastric space by imbricating the greater curvature wall with suture^7,8^. Previous studies by the same surgical team, showed that it is a feasible technique and has a mechanism of restriction like that of sleeve gastrectomy, albeit without any stapling or organ resection^8^. As there is no section of the stomach, less morbidity occurs, once there is no probability of stapling or anastomotic leaks. A meta-analysis that evaluated the effects of GP on obesity and type 2 diabetes mellitus concluded that this technique effectively leads to weight loss in the 1–2 years, with the pooled percentage of TWL ranging from 11–25%^12^. In the present study, an average TWL overall percentage of about 16% was observed 2–4 years after surgery.

The GP-FP may be considered an alternative for obese individuals with GERD. It is a procedure that does not use foreign bodies, such as bands, and potentially leads to lower costs, since there is no need for staplers and other materials, and to less perioperative morbidity and hospital stay^5,16^. Both Lee et al.^9^, in a series of 25 participants, and Talha et al.^17^, in a pioneering study including 18 patients, demonstrated comparable outcomes of GP-FP, with complete resolution of GERD-related symptoms and, respectively, 18.6 and 18.1% of TWL at one year postoperatively. Ospanov et al.^13^, in a comparison between fundoplication alone and GP-FP in individuals with mild and moderate obesity, observed similar outcomes in relation to GERD and a significantly higher weight loss after GP-FP. The present study demonstrated a comparable percentage of TWL at 2–4 years associated with complete resolution of esophageal symptoms at 1–2 and 2–4 years postoperatively. These findings were entirely consonant with the strongly significant reduction of the DeMeester score at pH monitoring and also with the improvement at esophagogastroscopy evaluation.

Considering the novelty of this procedure, it is imperative to acknowledge that the implementation of any technique may imply a decreasing complication rate over time according to the learning curve and skills of each surgical team. Talebpour et al.^16^ reported that in 70 individuals who underwent isolated GP, nausea and vomiting were the most common perioperative complications (91.4%), but major morbidity occurred in only 2.9%. Talha et al.^17^, in the first 18 cases of GP-FP, no major morbidity was observed. Ospanov et al.^13^ described two cases of bleeding after GP-FP, one requiring laparoscopic re-exploration. These studies included individuals with higher BMIs than those included in the current study. This, alongside the retrospective data collection of the present study (which does not favor the detection of minor morbidity), may help explain the somewhat more favorable outcomes of the current study. Considering the equally low perioperative morbidity usually observed in the currently standard bariatric operations, mainly sleeve gastrectomy and Roux-en-Y gastric bypass, the findings of the present study are encouraging^2,6^.

The current study has limitations worth mentioning. Its retrospective design can lead to lower quality data, as well as the varying postoperative time points at which the individuals were evaluated. In addition, the small sample associated with a very homogeneous study population does not allow for broader inferences or automatic extrapolation of results to other groups. There was a significant number of individuals with unavailable postoperative pH monitoring and endoscopic assessment data, factors that also influence negatively the data quality. The telephone survey method also interferes with the results, since it depends on the accuracy of the data provided by the participants and subjects them to the risk of social expectation bias in their responses. Further studies are needed to better assess the long-term benefits of this procedure, as well as a rigorous follow-up to verify the adopted lifestyle. It should also be emphasized that the operations included in this study were performed by a surgical team highly experienced in both esophageal and bariatric proceedings; in regard to the techniques evaluated, this team had passed through a lengthy learning curve in both Nissen operation and isolated GP at a high-volume center. Thus, the perioperative outcomes currently presented should not be considered entirely reproducible for teams without such expertise. Nevertheless, the findings of the present study allow us to conclude that it is an effective procedure that leads to symptomatic and endoscopic improvement of gastroesophageal reflux associated with significant weight loss in individuals with combined mild obesity and GERD, in addition to effectively producing a decrease in acid reflux assessed through pH monitoring.

## CONCLUSIONS

The GP-FP proved to be an effective and safe technique, leading to a significant and sustained weight loss as well as endoscopic and clinical improvement of GERD.
